# Meeting of Confederate Surgeons

**Published:** 1874-05

**Authors:** 


Elsewhere we publish the call for a meeting of the former Medical Officers of the Confederate States Army to be held in this city on the 20th of the present month.

The proposed gathering of Confederate Surgeons, we trust, will be largely attended, and that proper steps will be taken to rescue from oblivion the mass of facts which now lie scattered among the individual members of the staff, and which, but for such concerted action, would be forever lost. We hope and expect that the social feature of this re-union will be of the happiest possible character—that the friendships of the camp-fire will be revived again, and cemented firmly by association in patriotic and scientific work. Let all who can possibly do so, lay aside their home toil for a season and come to the Confederate Surgeons' re-union. Let us revive the memories of those long and dreary years of self-sacrifice, of toil and suffering which we patiently and willingly endured in behalf of the land which gave us birth, a cause which we fondly cherished, and one in which, though now a "lost cause," we find nothing of shame, but much of manly pride. Let us put upon record, as far as we can, the enduring recollection of our deeds, that we may help to build up the future history of this great struggle for our children and their descendants.

